# Deficits in Pre-attentive Processing of Spatial Location and Negative Symptoms in Subjects at Clinical High Risk for Schizophrenia

**DOI:** 10.3389/fpsyt.2020.629144

**Published:** 2021-02-02

**Authors:** Pejman Sehatpour, Michael Avissar, Joshua T. Kantrowitz, Cheryl M. Corcoran, Heloise M. De Baun, Gaurav H. Patel, Ragy R. Girgis, Gary Brucato, Javier Lopez-Calderon, Gail Silipo, Elisa Dias, Antigona Martinez, Daniel C. Javitt

**Affiliations:** ^1^College of Physicians and Surgeons, New York State Psychiatric Institute, Columbia University, New York, NY, United States; ^2^Schizophrenia Research Division, Nathan Kline Institute for Psychiatric Research, Orangeburg, NY, United States; ^3^Icahn School of Medicine at Mt. Sinai, New York, NY, United States; ^4^Centro de Investigaciones Médicas, Escuela de Medicina, Universidad de Talca, Talca, Chile

**Keywords:** auditory event-related potentials, mismatch negativity, location deviant, schizophrenia, clinical high-risk, fMRI, functional connectivity, auditory event related potential

## Abstract

Deficits in mismatch negativity (MMN) generation are among the best-established biomarkers for cognitive dysfunction in schizophrenia and predict conversion to schizophrenia (Sz) among individuals at symptomatic clinical high risk (CHR). Impairments in MMN index dysfunction at both subcortical and cortical components of the early auditory system. To date, the large majority of studies have been conducted using deviants that differ from preceding standards in either tonal frequency (pitch) or duration. By contrast, MMN to sound location deviation has been studied to only a limited degree in Sz and has not previously been examined in CHR populations. Here, we evaluated location MMN across Sz and CHR using an optimized, multi-deviant pattern that included a location-deviant, as defined using interaural time delay (ITD) stimuli along with pitch, duration, frequency modulation (FM) and intensity deviants in a sample of 42 Sz, 33 CHR and 28 healthy control (HC) subjects. In addition, we obtained resting state functional connectivity (rsfMRI) on CHR subjects. Sz showed impaired MMN performance across all deviant types, along with strong correlation between MMN deficits and impaired neurocognitive function. In this sample of largely non-converting CHR subjects, no deficits were observed in either pitch or duration MMN. By contrast, CHR subjects showed significant impairments in location MMN generation particularly over right hemisphere and significant correlation between impaired location MMN and negative symptoms including deterioration of role function. In addition, significant correlations were observed between location MMN and rsfMRI involving brainstem circuits. In general, location detection using ITD stimuli depends upon precise processing within midbrain regions and provides a rapid and robust reorientation of attention. Present findings reinforce the utility of MMN as a pre-attentive index of auditory cognitive dysfunction in Sz and suggest that location MMN may index brain circuits distinct from those indexed by other deviant types.

## Introduction

Deficits in early sensory processing represent a critical component of schizophrenia and contribute directly to poor functional outcome. For example, in the visual system, functional deficits starting in retina and involving primarily the magnocellular visual system contribute to impaired perceptual closure, face emotion recognition and reading ability. In the auditory system, deficits in the ability to process low-level sensory information such as pitch and duration correlate with impaired prosodic processing and social cognition. In addition to providing key targets for intervention, sensory-level deficits may also be useful for probing basic neurophysiological processes underlying symptom generation and impaired cognitive function in schizophrenia [rev. in (1, 2)].

Thus, for example, recent studies have shown that in the visual system, differential patterns of sensory event-related potentials (ERP) may differentiate between schizophrenia (Sz) and autism-spectrum disorder (ASD) subjects (3), and, in Sz, motion processing deficits may be present even prior to disease onset (4). In the auditory system, mismatch negativity (MMN) has proven among the most effective tools both for analyzing neurophysiological mechanisms underlying impaired auditory function in Sz and for early detection of individuals at clinical high-risk (CHR) for Sz. MMN is elicited by simple auditory stimuli that differ physically or conceptually from preceding repetitive standards [rev. in (5)].

Deficits in MMN in schizophrenia were first demonstrated ~30 years ago to simple duration and pitch deviant stimuli and have been replicated extensively since that time with a mean effect-size of 0.65 sd units across studies (6). Moreover, deficits correlate strongly with impaired cognitive function and negative symptoms, which, in turn, predict impaired functional outcome (7). Primary generators for MMN are localized to supratemporal auditory cortex, leading to a characteristic scalp distribution with maximal amplitude over the frontocentral scalp and inversion of polarity at electrodes below the orientation of superior temporal plane.

Deficits in MMN generation in Sz are associated with impaired activation of superior temporal auditory cortex as reflected in both ERP source localization (8, 9) and fMRI (10, 11) studies, as well as reduced activation of subcortical regions such as inferior colliculus and thalamus (12). During MMN paradigms, activation is also observed within prefrontal cortex and a salience network that includes insula and anterior cingulate cortex (9, 11, 13). By contrast, MMN is associated with de-activation of visual and dorsal attention systems (11). In resting state functional connectivity (rsFC) fMRI analyses, MMN deficits correlate with reduced rsFC both within auditory regions, and between auditory cortex and somato-motor, dorsal attention, ventral attention and default networks (14), as defined using a 7-network parcellation scheme (15). The present study performs similar rsFC analyses using a more recently developed network parcellation approach (16). We combined this with a subcortical volumetric parcellation from FreeSurfer (17, 18) that includes separate regions of interest (ROIs) for brainstem and thalamus.

Sz-like deficits in MMN generation are induced by N-methyl-D-aspartate receptor (NMDAR) antagonists such as ketamine across rodent (19), monkey (20, 21) and human (22) models, supporting translational use of MMN in support of glutamatergic models of Sz. As opposed to alternative biomarkers such as the auditory steady-state response that are reflected primarily in alteration of high-(gamma) frequency oscillations and reflect dysfunction primarily of parvalbumin (PV) interneuron-related circuits, MMN generation is associated with alterations primarily in theta frequency responses across species, suggesting primary involvement of somatostatin (SOM) interneurons (21, 23).

Within the context of an overall reduction in MMN in Sz, differential impairments and correlation patterns may be observed across MMN types. Thus, whereas deficits in duration MMN are observed across Sz populations generally, frequency MMN deficits appear to index a subgroup of poor outcome patients with neurophysiological abnormalities extending even into subcortical components of the auditory pathway such as medial geniculate nucleus (MGN) or inferior colliculus (IC) (12, 14, 24). The poorer outcome of individuals with frequency+duration vs. duration MMN alone deficits may reflect the critical role that pitch discrimination plays in daily life, such as detection of emotion or attitude based on tone of voice (2, 25–28) or phonetic aspects of reading (29). In general, MMN deficits are similar to or smaller than complex vs. simple deviants (6), supporting the importance of bottom-up mechanisms.

To date, an extensive literature has accumulated regarding MMN to pitch and duration deviants in both Sz and CHR patients relative to healthy controls (HC). By contrast, less information is available regarding other low-level deviant types, in particular, location MMN. As with other low-level auditory features, infrequent deviation in stimulus location elicits an MMN that is maximal over frontocentral scalp, suggesting generators located in posterior STG (30–32) and that increases progressively within increasing stimulus deviance (33).

In general, horizontal sound localization in space relies on two sets of binaural cues: first, the differences in the time of arrival at the two ears, termed interaural time difference (ITD) or the closely related interaural phase difference (IPD), and second, the difference in intensity between the sound arriving at the two ears, which is termed either interaural level (ILD) or intensity (IID) difference. The location MMN system appears sensitive to both sets of localization cues, which are processed in parallel (34). A consistent additional finding is that location MMN has a shorter latency relative to other deviant types (35), consistent with ultrafast processing of stimulus location within subcortical auditory structures so that initial location deviance is observed as early as 20 ms following stimulation (36).

To date, a limited number of studies have investigated localization ability and location MMN generation in Sz (37, 38). In two prior studies, we have observed intercorrelations between impaired localization ability and severity of both thought disorder and auditory verbal hallucinations (AVH), such that severity of AVH correlated with paradoxically preserved MMN to right hemifield stimuli and severity of thought disorder correlated significantly with impaired spatial location ability to right hemifield stimuli (39, 40). However, to our knowledge, integrity of location MMN has not been previously investigated in CHR individuals. Spatial processing plays an important but largely unconscious role in everyday life. For example, spatial separation is one of the primary mechanisms by which individuals identify “objects” in space and differentiate information coming from a single source vs. background (33). Here, we investigated location MMN generation relative to that induced by other deviant types in independent groups of Sz and CHR subjects, using ITD-stimuli to simulate Left-going and Right-going stimuli relative to a central location.

Based upon our prior studies, we hypothesized that location MMN generation would be impaired in Sz and that impairments would correlate with severity of thought disorder and AVH. In CHR, we hypothesized that MMN amplitude would be reduced as well and would correlate with overall function. Key additional questions were to investigate (1) the spectral properties of location MMN relative to other MMN types using time-frequency (TF) analyses; and (2) the degree to which inclusion of location deviants within a multi-deviant paradigm affects the overall pattern of result. Finally, in general, standards within the MMN paradigm are analyzed to only a limited degree, but we have recently demonstrated impaired generation in Sz using TF analyses. Here, we investigated integrity of standard processing in CHR as well.

## Methods

### Participants

This study included patients with schizophrenia who met DSM-5 criteria (*N* = 42), CHR (*N* = 34) who were diagnosed with the Structured Interview for Psychosis-Risk Syndromes, and age-matched healthy volunteers (*N* = 28). The clinical population was recruited from New York State Psychiatric Institute at Columbia University and the Nathan Kline Institute for Psychiatric Research. The healthy controls (HCs) were recruited from the surrounding communities. The study was approved by the institutional review boards of the respective institutes, and written informed consent was obtained from all study subjects. Individuals with organic brain disorders, IQ < 70, past drug or alcohol dependence, current drug or alcohol abuse, or hearing/vision impairments were excluded. Additionally, attenuated psychosis symptoms could not occur solely in the context of substance use or withdrawal or be better accounted for by another disorder including a medical condition known to affect the CNS. Of the 21 CHR subjects who completed 2-yr follow-up, 3 converted to Sz within the time frame of the study (14.7%), while others remain within the follow up period (*n* = 10). Thirteen of the CHR subjects were receiving low-dose antipsychotic medication at the time of testing.

### Symptoms and Neuropsychological Measures

Psychiatric symptoms were evaluated using the Positive and Negative Syndrome Scale (PANSS). Attenuated psychosis symptoms were assessed using the Scale of Prodromal Symptoms. General neuropsychological function was assessed with the MATRICS (Measurement and Treatment Research to Improve Cognition in Schizophrenia) Consensus Cognitive Battery (MCCB) (see Table 1).

**Table 1:** Demographics.

	**Schizophrenia**	**Clinical High Risk**	**Control**
	**(*N* = 42)**	**(*N* = 33)**	**(*N* = 28)**
Age	34.9 (8.2)	22.1 (4.3)	34.0 (13.0)
Gender (F/M/O)	7/35	15/16/2	11/17
CPZ Equiv.	540.9 (782.0)	42.6 (87.6)	–
PANSS/SOPS (positive)	16.1 (5.3)	13.8 (3.4)	–
PANSS/SOPS (negative)	16.6 (4.1)	14.4 (7.7)	–
PANSS/SOPS (general)	33.4 (8.7)	10.9 (5.5)	–
SOPS (disorganization)	–	8.7 (4.6)	–
PANSS/SOPS (total)	66.1 (14.4)	47.8 (18.7)	–
Tone Matching	80.7% (13.9%)	90.8% (10.3%)	88.6% (10.2%)
MATRICS Mean T-score	38.3 (10.2)	44.1 (6.1)	48.6 (6.8)

### Data Acquisition and Analysis Procedure

#### Stimuli and Task

Auditory stimuli consisted of a sequence of tones presented in random order with a stimulus onset asynchrony (SOA) of 500 ms. Standard stimuli (45% sequential probability) were harmonic tones composed of three superimposed sinusoids (500, 1,000, and 1,500 Hz) 100 ms in duration with 5-ms rise/fall time presented at ~85 dB.

Six deviants were used i.e., pitch, duration and intensity (10% probability each) were 10% higher in pitch, 50 ms longer in duration, 45% lower in intensity, respectively, and frequency modulated (at 2 Hz with modulation index of 300) deviant (10% probability). All the above tones were presented binaurally with apparent location in the center midline. Two location deviants were included (7.5% probability each) that gave the percept of stimulus movement to the left vs. right hemifield based on an interaural delay time of 700 μs between ears in the appropriate direction. Seven runs of 5 min each (600 stimuli/run) were presented as the subjects listened to the tones while watching a silent movie as a distractor.

#### EEG Data Acquisition and Analysis

Continuous EEG was acquired through Brain Vision Brainamp MR Plus amplifier system using 64 scalp electrodes (10-10 system), impedances <5 kΩ, referenced to the FCz electrode, bandpass filtered from 0.05 to 100 Hz, and digitized at 500 Hz. Data were re-referenced to linked-mastoid reference and analyzed offline using MATLAB software, version 2017a (MathWorks) and EEGLAB, ERPLAB toolboxes. An independent component analysis (ICA) was performed for removal of blink-related artifacts. Epochs of 1,000 ms prior to the onset of each stimulus to 1,000 ms post stimulus were derived. Epochs with amplitudes exceeding ±100 μV at any electrode were also excluded. On average, 14% of trials were excluded in the schizophrenia group, 24% in the CHR group and 19% in the control group.

ERPs were obtained by time locking to the onset of all stimuli and averaging across trials baselined from −300 to 0 ms, using a notch filter at 60 Hz, a high pass filter at 0.1 Hz, and a low pass filter at 80 Hz. Time-frequency (TF) evoked amplitude measures were obtained by convolving the time-domain averaged event-related potentials with five-cycle Morlet wavelets over the entire 2,000-ms window of the epochs. TF data were derived at each time point for a frequency range of 0.5–50 Hz at 1 Hz increments. Likewise, single-trial TF transformations were derived to compute baseline-corrected single-trial power and inter-trial phase locking (ITPL/ITC). ITC reflects the consistency of spectral response across repeated trials ranging from 0 (no consistency) to 1 (perfect consistency). In general, changes in ITC in the absence of alterations in spectral power are thought to reflect stimulus induced phase reset of ongoing oscillatory activity. Three scalp ROIs namely frontocentral (F1, Fz, F2, FC1, FC2), left hemisphere (FC5, FC3, C5, C3) and right hemisphere (FC4, FC6, C4, C6) were derived by averaging the signal from electrodes in each ROI. The frequency ranges of interest were derived by averaging the TF data within each frequency range as theta 4–8 Hz, alpha 8–12 Hz, and beta 12–24 Hz.

### Resting State Functional Connectivity

Resting state fMRI data were obtained for CHR subjects only as part of a larger study.

#### fMRI Acquisition and Preprocessing

Twenty-three CHRs underwent anatomical and Resting-State fMRI (RS-fMRI) scans. Two to four resting state scans were collected on a 3T GE Scanner for each participant. Each of the two to four scans lasted 5 min and 30 s. Structural T1 and T2 (0.8 mm isotropic), multiband (MB) fMRI (2 mm isotropic, TR = 900 ms, MB factor 6), and distortion correction scans (B0 fieldmaps) were acquired as required for use with the Human Connectome Project (HCP) processing pipelines. The HCP pipeline performs standard preprocessing procedures (alignment to individual's anatomical data, movement correction, distortion correction, and atlas alignment), along with surface-based extraction and surface atlas alignment of gray matter voxels to improve co-registration of functional maps between individuals and with standard surface atlases (41).

#### fMRI Analysis

Regions of interest (ROIs) were based on the atlas by Gordon et al. (16) that parcellates the cortical surface using resting state functional connectivity (rsFC) data. We merged all parcels belonging to the same network into single ROIs so that each ROI represented one network. We also included volumetric subcortical parcels used in segmentation procedures in FreeSurfer (17, 18). We analyzed twelve cortical networks along with subcortical thalamus and brainstem regions.

Standard post-processing procedures adapted from Power et al. were used to minimize movement-related artifacts (42) with details described in a prior study (43), with a framewise displacement (FD) threshold of 0.2 mm for frame censoring. Resting state functional connectivity analyses were then performed by correlating the cleaned time-course of each ROI-pair (Pearson's correlations) within each participant.

### Statistical Analyses

Primary between-group comparisons were analyzed using repeated measures analysis of variance. For Sz, all controls were used as comparison. For CHR analyses, only HC who fell within the predefined age range of the CHR subjects (13–30 yo) were included in the control group.

Between-group analyses were conducted on time-domain ERP measures using univariate or repeated measures ANOVA with group and sex as factors and site and age as covariates where appropriate. For each significant between-group effect, evoked-power analyses were then used to assess TF content of the component, and source of the between-group difference. Finally, single-trial analyses were used to assess the relative contribution of ITC and power to the evoked power differences.

Correlational analyses assessed the interrelationship between location MMN and symptoms in both Sz and CHR populations using univariate correlations. Within the CHR group, correlations with network rsFC measures were assessed using multivariate stepwise regression across cortical and subcortical regions. Correlation strength was evaluated based on partial correlation (r_p_) between dependent and independent variables.

## Results

### Location MMN

**Figure 1:**
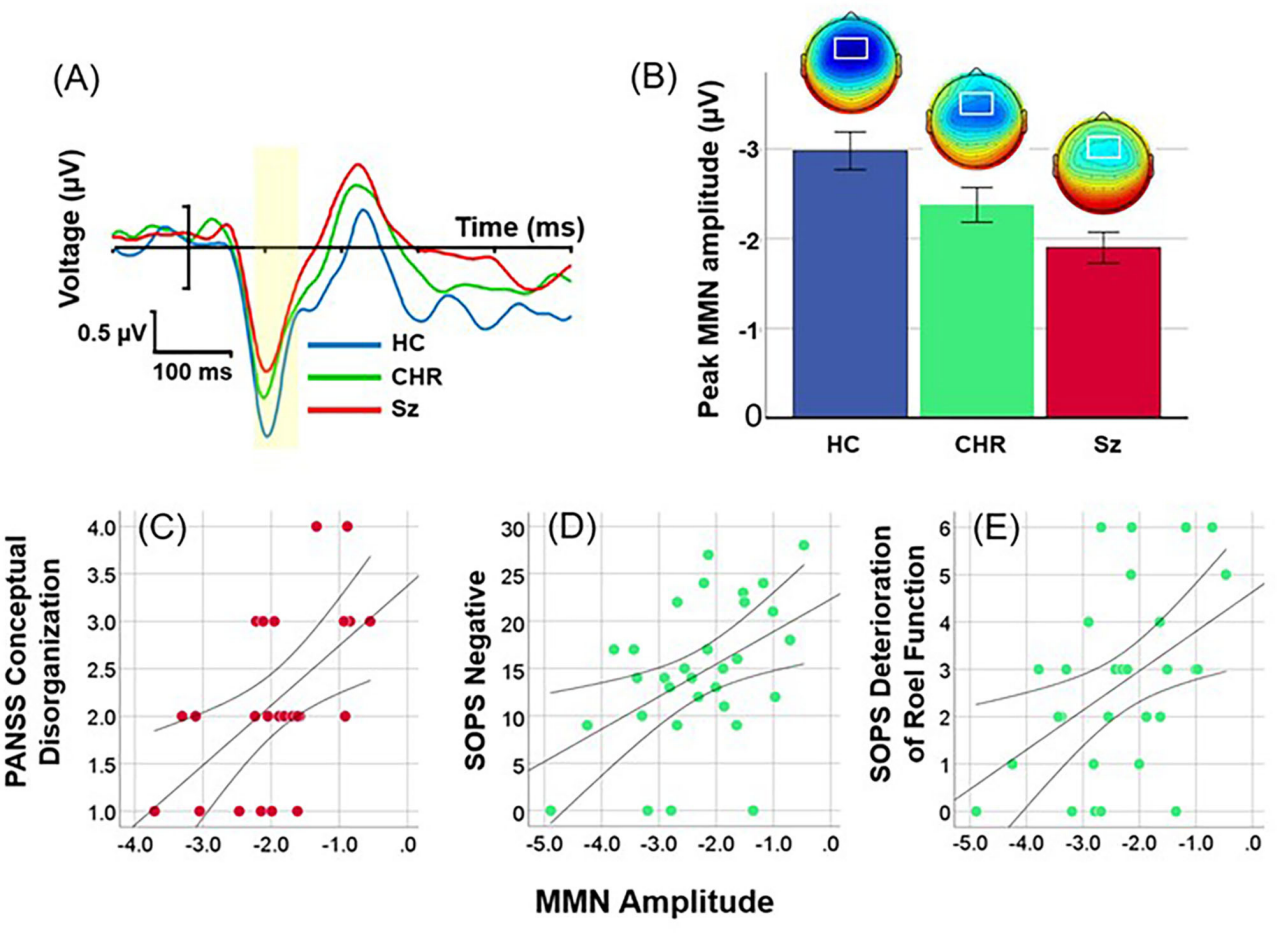
Event-related potential (ERP) difference waves to location deviants across healthy controls (HC), Clinical High Risk (CHR) and Schizophrenia (Sz). **(A)** Time-domain waveforms by group. The peak latency window is shown in yellow. **(B)** Mean amplitudes (± sem) and scalp topographies by group. **(C)** Correlation between location MMN and conceptual disorganization in Sz patients. **(D,E)** Correlation between SOPS Negative Symptom factor and Deterioration of Role Function item in CHR.

Location of MMN was maximal over frontocentral scalp, with latency of 112 ms across groups (Figure 1A). Overall amplitude of location MMN over the fronto-central scalp was significantly different across groups (F_(2, 99)_ = 7.89, *p* = 0.001), with significant deficits for Sz (F_(1, 67)_=17.3, *p* < 0.0001, *d* = 1.1) and CHR (F_(1, 46)_ = 4.59, *p* = 0.037, *d* = 0.63) groups independently (Figure 1B). The between-group difference remained strongly significant even following control for age, sex and study site (F_(2, 93)_ = 8.19, *p* = 0.001). No significant effects were observed for age (*p* = 0.48), sex (*p* = 0.1) or site (*p* = 0.62), or for interactions among these measures.

When analyses were performed by direction using combined left and right ROIs, there was again a highly significant difference across groups (F_(2, 99)_ = 7.93, *p* = 0.001). Neither the main effect of direction (F_(1, 99)_ = 1.95, *p* = 0.17) nor direction X group interactions (F_(2, 99)_ = 0.23, *p* = 0.8) were significant.

#### Correlation With Symptoms and Neuropsychological Function

As predicted, in Sz (*n* = 37), amplitude of location MMN over right hemisphere correlated with severity of conceptual disorganization (*r* = 0.38, *p* = 0.022) (Figure 1C). In addition, significant correlations were observed with PANSS Total symptoms (*r* = 0.33, *r* = 0.049), Somatic concern (*r* = 0.47, *p* = 0.003), Delusions (*r* = 0.35, *p* = 0.036), Passive/Apathetic social withdrawal (*r* = 0.39, *p* = 0.019) and Preoccupation (*r* = 0.38, *p* = 0.022).

In CHR (*n* = 31), amplitude of location MMN over right hemisphere correlated significantly with Negative (*r* = 0.46, *p* = 0.009) (Figure 1D) and General (*r* = 0.45, *p* = 0.012) factor scores along with the specific symptoms within the Negative factor of Deterioration in Role Function (*r* = 0.46, *p* = 0.009) (Figure 1E), Decreased Experience of Emotion and Self (*r* = 0.40, *p* = 0.024), Social Isolation (*r* = 0.44, *p* = 0.013), and Avolition (*r* = 0.43, *p* = 0.017); and specific symptoms within the General factor of Dysphoric Mood (*r* = 0.53, *p* = 0.003) and Impaired Tolerance to Normal Stress (*r* = 0.43, *p* = 0.015).

#### Correlation With Cognition

In Sz (*n* = 24) impaired location MMN generation over right hemisphere correlated significantly with reduced MCCB total score (*r* = −0.56, *p* = 0.004) as well as Speed of Processing (*r* = −0.51, *p* = 0.011), Verbal Learning (*r* = −0.51, *p* = 0.01), Working Memory (*r* = −0.45, *p* = 0.027) and Visual Learning (*r* = −0.54, *p* = 0.007). No significant correlations were observed in CHR subjects.

#### Time-Frequency

**Figure 2:**
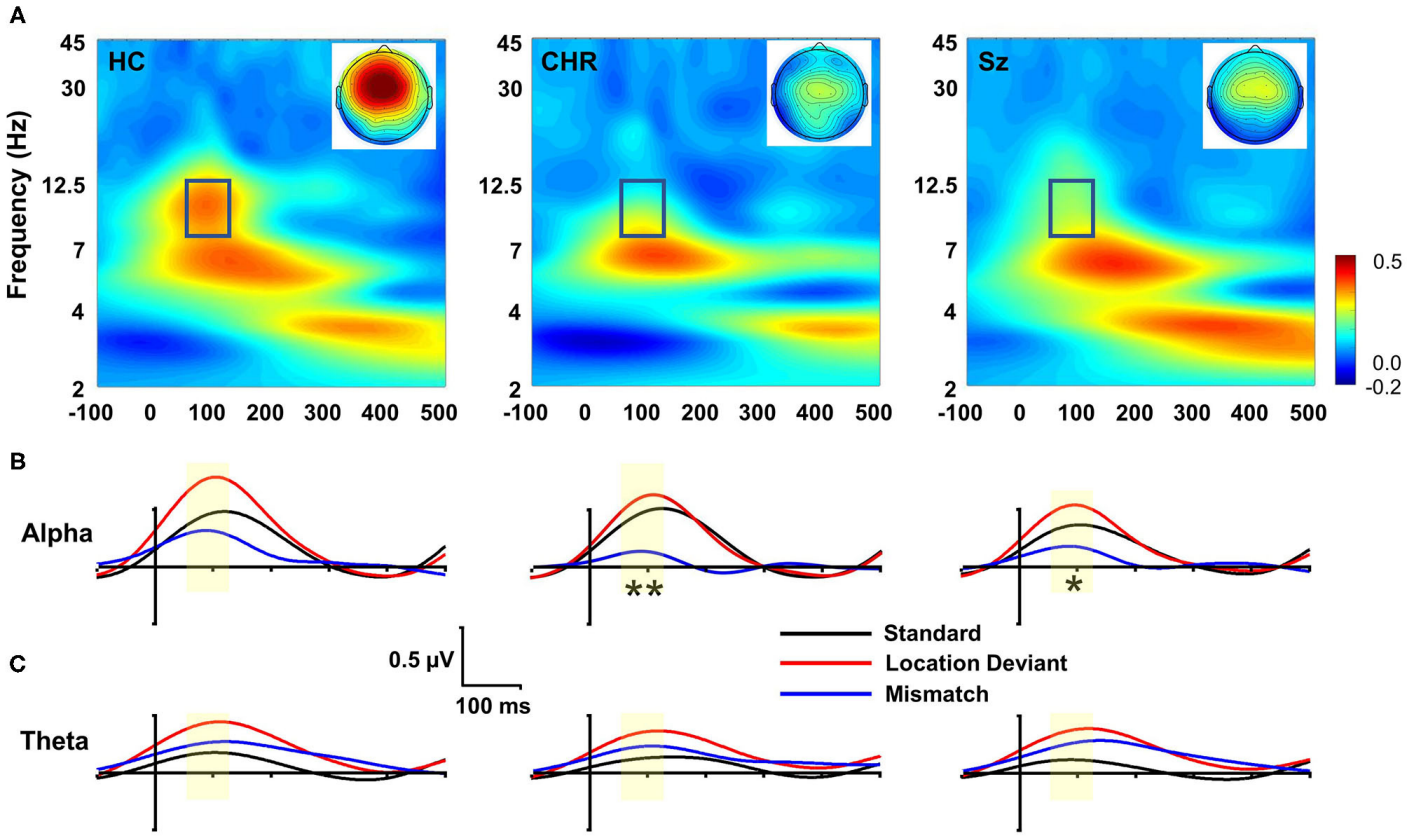
Time-frequency (TF) analysis of location MMN deficits. **(A)** TF plots of location MMN by group. Box region shows alpha response interval. Inset: Scalp topographies with the alpha integration window. **(B)** Alpha-band evoked power by group over time for standards, deviants and mismatch difference wave. **(C)** Theta-band evoked response. *p < 0.05; **p < 0.01.

In evoked power analyses, location MMN was associated with activity in both the alpha and theta frequency ranges (Figure 2A). Alpha activity was limited to the 55–125 ms range and was concentrated at frontocentral electrodes. Evoked alpha power differed significantly across groups (F_(2, 99)_ = 3.91, *p* = 0.023) (Figure 2B), whereas evoked theta power did not (F_(2, 99)_ = 0.67, *p* = 0.6) (Figure 2C). Furthermore, in ANCOVA, amplitude of MMN covaried significantly with amplitude of evoked alpha power (F_(2, 97)_ = 5.94, *p* = 0.004) but not evoked theta power (F_(2, 97)_ = 0.003, *p* = 0.96) above effects of group.

In single trial analyses, deficits in alpha ITC were also significantly different between groups (F_(2, 99)_ = 3.62, *p* = 0.03) and covaried strongly with deficits in location MMN (F_(1, 97)_ = 379.7, *p* < 0.0001) over and above the effect of group. Once the ITC deficits were taken into account, the between-group difference in evoked power was no longer significant (F_(2, 97)_ = 0.35, *p* = 0.7). No significant differences were observed for changes in total power (F_(2, 99)_ = 2.33, *p* = 0.1), and total power did not co-vary significantly with evoked power (F_(1, 97)_ = 1.13, *p* = 0.29). By contrast to alterations in alpha-related activity, no significant difference was observed in theta evoked power across groups (F_(2, 99)_ = 0.57, *p* = 0.6).

### Other MMN

**Figure 3:**
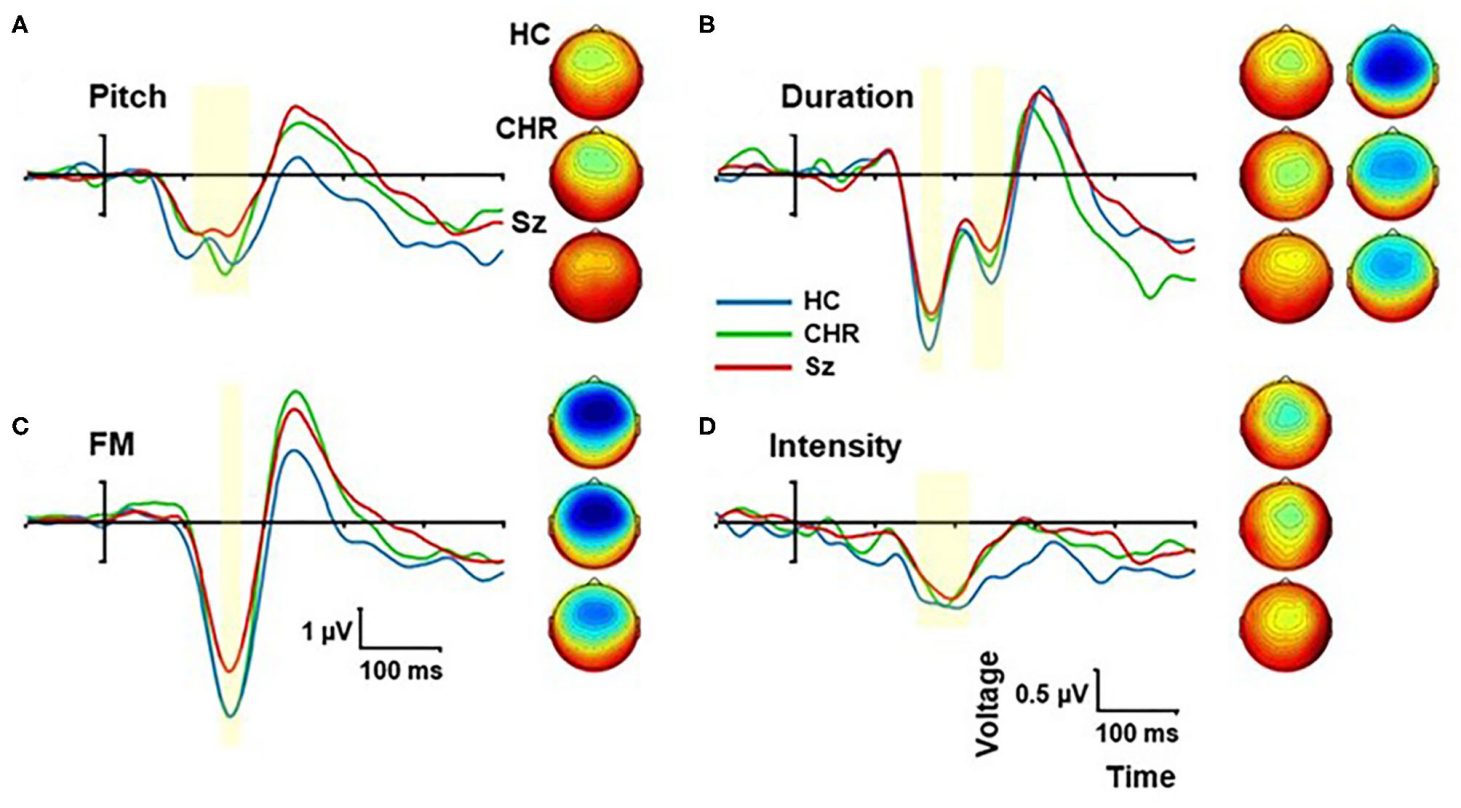
Event-related potential (ERP) difference wave to Pitch **(A)**, Duration **(B)**, Frequency modulation (FM) **(C)**, and Intensity **(D)** deviants by group. The peak latency window is shown by yellow shading. Scalp topographies are show by group for each deviant type within the peak window.

In addition to location MMN, we incorporated pitch, duration, intensity, and FM deviants into the multi-deviant sequence (Figure 3). Across all deviants, there was a significant main effect of group (F_(2, 99)_ = 4.78, *p* = 0.01) that remained significant when analyses were controlled for age, sex and study site (F_(2, 93)_ = 4.36, *p* = 0.015). The effect of deviant type was highly significant (F_(3, 97)_ = 110.7, *p* < 0.0001) but it did not interact significantly with group (F_(6, 196)_ = 1.53, *p* = 0.1). In pairwise tests, the reductions were significant for Sz vs. HC (F_(1, 67)_ = 7.85, *p* = 0.007, *d* = 0.68) whereas the difference between CHR and similar-age HC was not significant (F_(1, 46)_ = 0.17, *p* = 0.7, *d* = 0.12).

When the deviant types were analyzed independently, we observed significant differences between Sz and HC for frequency (F_(1, 67)_ = 10.1, *p* = 0.002, *d* = 0.78), FM (F_(1, 67)_ = 6.93, *p* = 0.011, *d* = 0.64) and duration (F_(1, 67)_ = 4.47, *p* = 0.038, *d* = 0.52) deviants, whereas the difference in intensity MMN was not statistically reliable (F_(1, 67)_ = 0.93, *p* = 0.34, *d* = 0.23).

#### Correlation With Symptoms

For Sz subjects, no correlation with symptoms was observed. For CHR, the mean MMN amplitude across the 4 deviant types (pitch, duration, intensity, FM) correlated with the severity of the Negative symptom factor score (*r* = 0.41, *p* = 0.023), especially Deterioration in Role Function (*r* = 0.47, *p* = 0.008), Decreased Expression of Emotion (*r* = 0.41, *p* = 0.02) and Avolition (*r* = 0.37, *p* = 0.04). However, these correlations were not significant for any of the MMN types independently.

#### Correlation With Cognition

For Sz subjects, impaired MMN generation to non-location deviants as a group correlated with reduced MCCB total score across domains (*r* = −0.56, *p* = 0.005) as well as Speed of Processing (*r* = −0.63, *p* = 0.001), Verbal learning (*r* = −0.52, *p* = 0.009) and Working Memory (*r* = −0.44, *p* = 0.033). Significant correlations were observed between MCCB total score and duration (*r* = −0.50, *p* = 0.014), intensity (*r* = −0.49, *p* = 0.015), and FM (*r* = −0.53, *p* = 0.008) deviants independently, although not for pitch deviants (*r* = −0.18, *p* = 0.41). Deficits in tone matching (*n* = 30) correlated specifically with impaired pitch MMN (*r* = −0.38, *p* = 0.037), but not to other MMN types. In CHR subjects (*n* = 17), no significant correlations with cognitive function were observed.

### Standard

**Figure 4:**
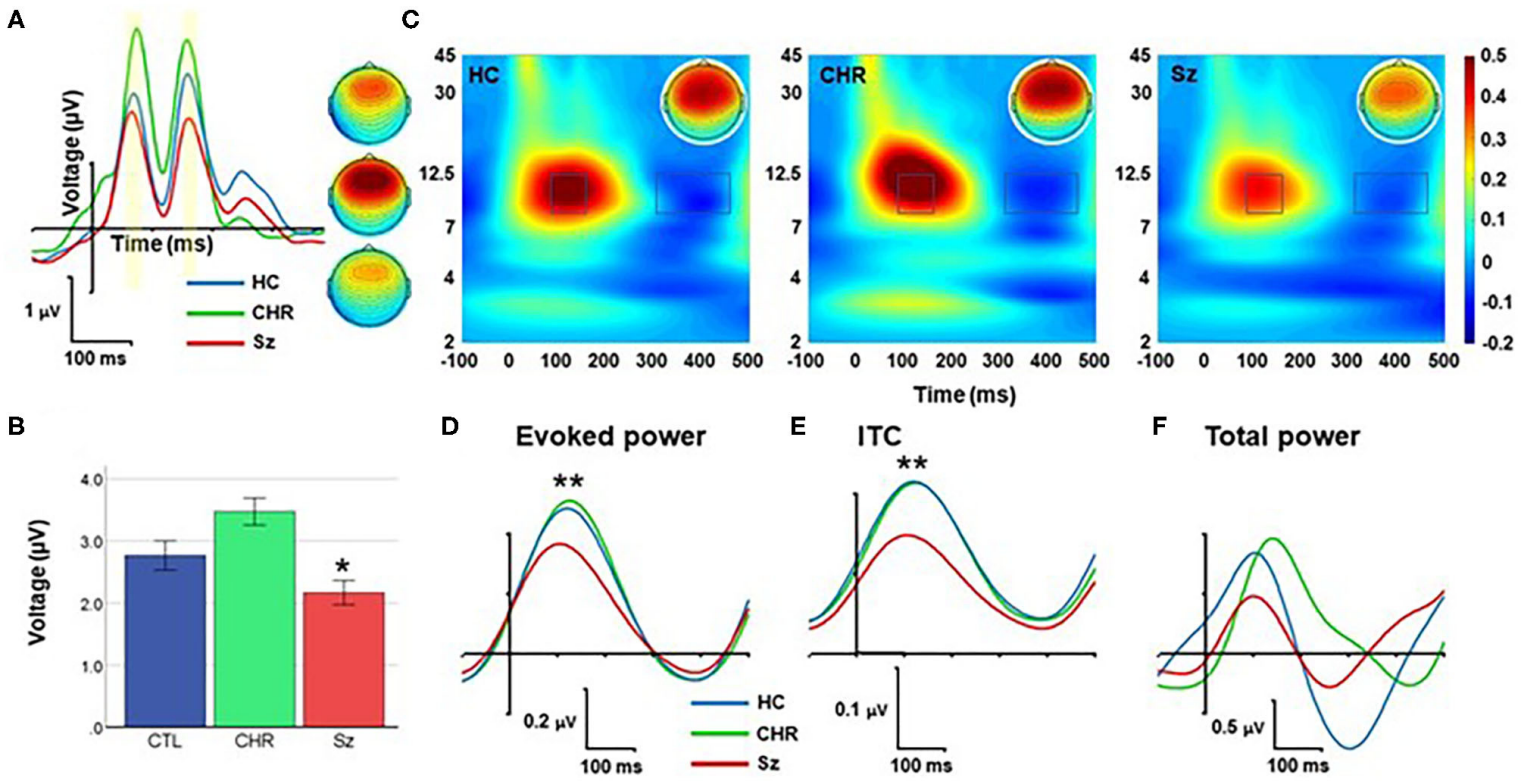
Response to standards. **(A)** ERP waveforms and scalp topographies by group. **(B)** Mean amplitude across each and late peaks. **(C)** Time-frequency (TF) plots. **(D–F)** Alpha-band responses over time for evoked power **(D)**, Intertrial coherence (ITC) **(E)**, and total power **(F)**. *p < 0.05; **p < 0.01.

Response to standard stimuli (Figures 4A,B) was significantly different across groups (F_(2, 99)_ = 9.98, *p* < 0.0001). The differences between Sz and HC subjects were also statistically reliable (F_(1, 67)_ = 4.79, *p* = 0.032). By contrast, no significant difference was observed for amplitude of the response to standards between CHR and similar-age controls (F_(1, 46)_ = 0.40, *p* = 0.53).

In TF analyses, the response to standards fell primarily within the alpha frequency range and was characterized by an initial increase during the 50–250 ms range followed by a suppression during the 350–450 ms range (Figure 4C). The magnitude of the initial increase (110–180 ms) was significantly different across groups (F_(2, 99)_ = 5.94, *p* = 0.004), and for Sz vs. HC independently (F_(1, 67)_ = 8.77, *p* = 0.004) (Figure 4D). Similarly, alpha ITC differed significantly across groups (F_(2, 99)_ = 10.1, *p* < 0.001) (Figure 4E) whereas single-trial alpha power was not significantly different (F_(2, 99)_ = 0.78, *p* = 0.46) (Figure 4F).

No significant correlations with symptoms were observed for either the Sz or CHR groups.

### MMN Latencies

As opposed to MMN amplitudes, no significant differences were observed in MMN latencies across groups either across deviant types (F_(2, 99)_ = 0.44, *p* = 0.65) or for any of the deviant types independently (all *p* > 0.2) (Table 2). As expected, there was a highly significant latency variation by deviant type (F_(4, 96)_ = 85.4, *p* < 0.0001) but no significant interaction with group (F_(8, 192)_ = 147, *p* = 0.17). The order of latencies was location < pitch < FM < duration < intensity, with significant differences between in repeated contrast testing (all *p* < 0.001).

**Table 2:** ERP latencies.

**Group**	**CTL**		**Sz**		**CHR**	
**Deviant**	**Mean**	**sd**	**Mean**	**sd**	**Mean**	**sd**
Location	115.1	31.1	111.8	25.9	108.6	29.4
Pitch	152.2	37.7	137.7	34.9	146.4	32.7
Duration	172.7	14.2	170.1	14.0	169.7	14.0
Intensity	185.2	32.8	186.9	30.6	181.8	39.9
FM	154.7	16.9	159.2	10.5	160.0	11.0

### rsFC Analyses

**Figure 5:**
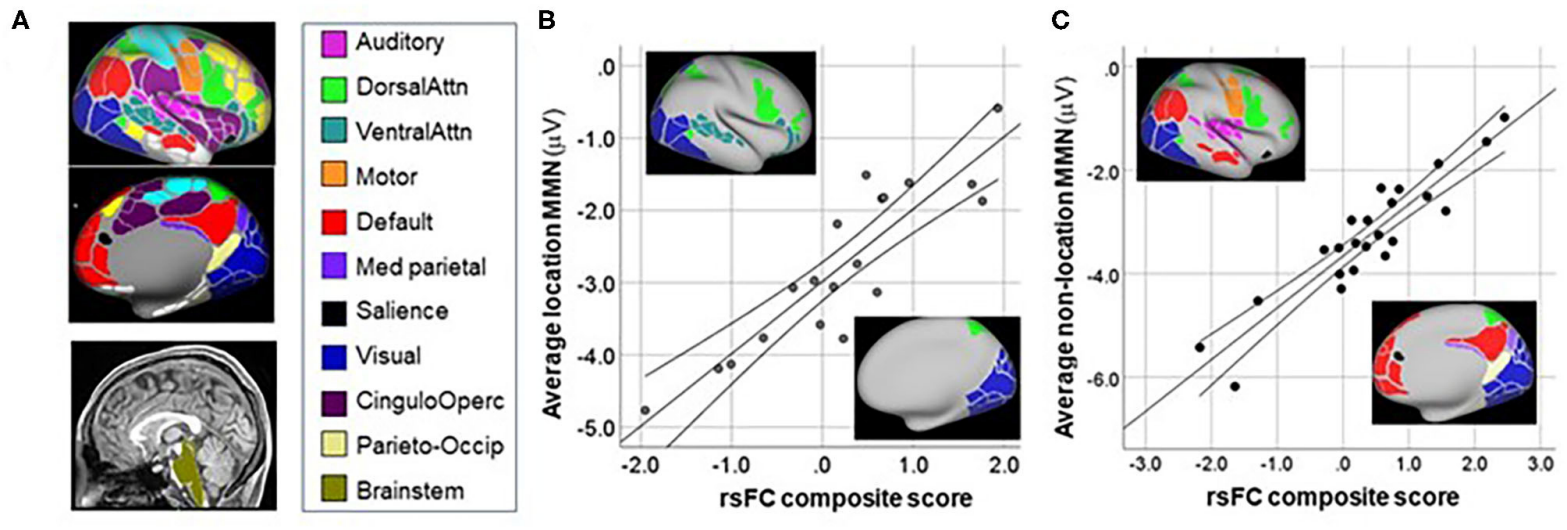
Correlation between MMN and resting-state functional connectivity (rsFC) networks. **(A)** Network maps from (16). **(B)** Correlation between mean MMN to location deviants and rsFC between networks shown in inset. **(C)** Correlation between mean MMN to non-location deviants and rsFC between networks shown in inset.

rsFC data were available for CHR patients only. Correlational analyses were performed using a pre-specified network-based parcellation approachh (16, 44) (Figure 5A).

For location MMN (Figure 5B), the primary negative correlations were between the Brainstem ROI and the Dorsal Attention system (r_p_ = 0.86, *p* < 0.0001), whereas positive correlations were observed between MMN and connectivity between Brainstem and both the Visual (r_p_ = 0.57, *p* = 0.016) and Ventral Attention (r_p_ = 0.85, *p* < 0.0001) systems. The overall model explained 78.5% of the variance (adj *R*^2^ = 0.785, *p* < 0.0001).

For combined MMN to non-location deviants (pitch, duration, FM, intensity) (Figure 5C) increased (i.e., more negative) MMN amplitude correlated significantly with increased rsFC between auditory and motor cortex (r_p_ = −0.57, *p* = 0.011), as well as between Medial Parietal and Default networks (r_p_ = 0.83, *p* < 0.0001). By contrast, an inverse relationship was observed between MMN amplitude and rsFC between Visual and Salience (r_p_ = 0.64, *p* = 0.003), Dorsal-Attention and Motor (r_p_ = 0.71, *p* = 0.001); and Parieto-Occipital and Default (r_p_ = 0.58, *p* = 0.01) networks. The overall model explained 80.8% of the variance (adj *R*^2^ = 0.808, *p* < 0.0001).

### Control Analyses

No significant correlations were observed with medication dose (CPZ equivalents) for any measure.

## Discussion

Deficits in MMN generation are among the most consistently observed neurophysiological abnormalities associated with Sz. They are also among the strongest predictors for conversion to psychosis among CHR. MMN is elicited by deviation in any of a number of physical or conceptual features, which are processed at varying levels of the neuro-axis from brainstem to secondary auditory regions [rev. in (5)].

In Sz and CHR, the vast majority of studies have been performed using pitch and duration deviants, which are easiest to generate and manipulate parametrically, and which show subtle differences in terms of sensitivity to patient type (6, 45). Here, we additionally evaluated integrity of location MMN generation, which has been studied in only a few prior Sz studies (37, 38, 40), and has not previously been investigated in CHR. As observed here (Table 2), location MMN has shorter onset latency than other deviant types, suggesting deviance detection even within subcortical components of the central auditory pathway. Consistent with this, changes in location are also more distracting than changes in other stimulus features (46).

Here, we confirmed prior findings of impaired location MMN generation in Sz, as well as our prior finding of association between localization impairment and severity of cognitive symptoms, especially conceptual disorganization. In addition, we demonstrate deficits in location MMN even among subjects who did not progress to Sz. In these subjects, deficits correlated highly with negative symptoms and particularly with deterioration in function, suggesting that location MMN may be sensitive to degenerative neural processes that predate illness onset and contribute to impaired function even in non-converting CHR subjects. The present study has potential etiological implications both for the CHR state and for individuals with existing psychosis.

### CHR

The initial studies of MMN in CHR individuals were first reported ~15 yrs ago (47), and multiple confirmation studies have been published over the past decade [e.g., (48–50)]. A meta-analysis performed in 2015 found mean effect sizes of *d* = 0.71 and *d* = 0.32 for duration and frequency MMN in CHR overall, but suggested that deficits may be driven primarily by the subgroup of patients who are truly prodromal for Sz (i.e., progress to psychosis) vs. those who meet APS criteria but do not develop full symptoms (51). Thus, a second meta-analysis observed an effect size deficit of *d* = 0.79 in converters, but only 0.17 in non-converters (52).

Since then, additional studies have been performed. For example, Atkinson et al. (53), performed a study in a large sample of 80 ultra high-risk individuals vs. 58 control subjects and did not observe between-group differences. Similarly, Hirt et al., (54) found no difference in duration and frequency MMN in at-risk individuals, despite significant MMN deficits in both early and late stage Sz. In general, the effect size deficit that we observed to the mean of “other” MMN (frequency, duration, intensity, FM), *d* = 0.12, is similar to the values observed in prior studies for APS individuals who were false, rather than true, prodromes for Sz.

Against this background, our present findings of significantly reduced location MMN (*d* = 0.63) even in a group of CHR subjects with a low conversion rate suggests that it may be differentially affected in CHR individuals relative to other forms of MMN, and thus may provide complementary information especially regarding causes of poor function even in non-converters. Indeed, deficits correlated highly with Negative symptoms (*p* = 0.006) and Deterioration of Role Function (*p* = 0.005) across the CHR state. These are important components of the CHR state independent of eventual conversion. To the extent that this finding can be replicated in larger studies, it argues for increased use during screening and increased etiological investigation into underlying mechanisms.

Consistent with the present study, a recent study also evaluated the P1 response to standards as part of a larger study on MMN (55). A small effect-size reduction (*d* = 0.1) was observed in true prodromes, whereas no significant deficits were found even in symptomatic non-converting subjects. In the present study, mean P1 amplitude was not significantly different across groups.

### Sz

As in CHR, the large majority of MMN studies in Sz have been performed with pitch and duration deviants. Deficits are found consistently in Sz (6) and correlate substantially with functional outcome and negative symptoms (7, 56). However, correlational effects have been of relatively small effect size (*r* = 0.2–0.3) and significant primarily due to the large sample sizes used in the prior studies.

Here, we report a moderate correlation (*r* = 0.46) for location MMN collapsed across deviant locations with PANSS derived Cognitive symptoms, especially conceptual disorganization. By contrast, the correlation between other deviant types and the Cognitive factor score of the PANSS was more modest and was not statistically significant in this sample (avg *r* = 0.28, *p* = 0.18). Nevertheless, larger samples are needed to directly compare effect-sizes across deviant types.

### Location MMN

MMN, in general, depends upon a process in which the auditory system maintains a short-duration (10–20 s) mnemonic template against which subsequent stimuli are compared. Detection of an acoustic violation (“mismatch”) between the template and the physical or conceptual features of the most recent stimulus triggers a neural mismatch process leading to enhanced current flow through open, unblocked NMDAR. This process of detecting a mismatch between the template and the incoming stimulus has increasingly been termed “prediction error.” However, it has become increasingly clear over recent years that such deviances may occur at multiple levels of the neuroaxis, depending upon the specific features involved.

Thus, for example, processes related to detection of frequency deviation may occur as early as 50 ms and may involve change detection within subcortical auditory structures including inferior colliculus (IC) and medial geniculate nucleus (MGN) (57). In Sz, MMN-related activation deficits are observed across all levels of the auditory system including IC, MGN, auditory and prefrontal cortex. Moreover, path analyses suggested that deficits propagated in a feed-forward fashion from subcortical to cortical nodes of the network (12), supporting bottom-up models of impaired MMN generation in Sz.

Against this backdrop, location is processed extremely early within the auditory pathway, and depends upon computations within midbrain auditory nuclei including the trapezoid body and the medial and lateral divisions of the superior olivary complex (MSO, LSO). This is especially true for computation ITD, which depends upon detection of extremely small delays on the order of 100–1,000 μs (58). These computations, which occur within the MSO, depend upon a unique computational circuit involving fast excitatory glutamatergic neurotransmission, mediated at GluA4-type AMPA receptors, and fast local inhibitory processes mediated at strychnine-sensitive glycine receptors. In parallel, ILD differences are computed primarily in the LSO (59). Together, MSO and LSO provide primary input into IC, which further integrates location and frequency information (58, 59). Consistent with this early computation, location MMN latency is significantly shorter than that of other deviant types (35, 36), as observed here. Moreover, we have observed that location MMN reflected activity primarily in the alpha, rather than theta, frequency range, and thus may represent involvement primarily of auditory thalamocortical projections (14).

There are also unique features related to location MMN processing at the cortical level. In the majority of mammals, including non-human primates, spatial localization is processed bilaterally by auditory cortex, such that each hemisphere processes sound coming from the opposite hemifield (60). In humans, spatial deviance-related activity is observed to location deviants within the medial superior temporal plane bilaterally (31). By contrast, because left auditory regions have been recruited in support of language processing, behavioral sound localization in humans is processed primarily by right auditory cortex, and is affected selectively by right-sided auditory lesions (61, 62). Thus, at the cortical level, impaired localization ability in Sz may selectively index right auditory dysfunction.

Integrity of location MMN has been studied in Sz to only a limited degree. An initial study of location MMN generation in Sz explored both ITD and ILD contributions, using stimuli that differed between ears by 700 μs or 16 dB, respectively. Both MMN and behavioral localization deficits were observed only with the ITD rather than the ILD cue (37). Furthermore, MMN deficits correlated with impaired ability to differentiate “signal” and “noise” sources in space (63), supporting behavioral relevance. A more recent study, however, found no significant MMN deficit to 800 μs ITD deviants in Sz when these were intermixed with other deviants in an optimal, multi-feature paradigm. However, the study also did not find deficits in other MMN types (38).

We have previously investigated localization ability in Sz in two prior studies. In the first (39), we found a deficit to location deviants overall, but more pronounced to Left-going deviants especially at intermediate locations (−30 to −45 degrees). In a follow-up study (40), we again observed impaired localization ability along with a significant correlation between positive thought disorder and localization ability within the Right hemi-field. We also observed reduced MMN amplitude overall as well as to Left hemifield deviants independently. In addition, we again observed impaired localization ability along with a significant correlation between positive thought disorder and localization ability within the Right hemi-field. AVH severity was associated with relatively preserved location MMN amplitude to deviant stimuli presented in the Right hemifield relative to a midline standard, even in the face of deficits to stimuli presented within the Left hemifield.

Here, with stimuli that were easier to administer but provided a less precise spatial percept, we found deficits most prominently across both directions and both left and right scalp regions (main effect *p* < 0.0001), but nevertheless did observe a significant 3-way group X direction X hemisphere interaction (*p* < 0.05), with larger deficits for Left-going (*p* < 0.0001) than Right-going (*p* < 0.003) deviants. Overall, however, much of the inner structure of the location MMN information was lost using simulated vs. free-field presentation of local deviance, suggesting need for complementary utilization of the two approaches.

In the present study, we observed a significant correlation between thought disorder as reflected in the conceptual disorganization item of the PANSS but did not observe a significant correlation between severity of AVH and MMN generation. A key reason may be that the use of ITD stimuli does not give an exact location percept. In our prior study, correlations to MMN were only observed over extreme right hemi-field locations (60, 90°). In the present study, ITD-based stimuli were experienced as coming vaguely from Left or Right hemi-fields but not from a specific location. In addition, in the present study we used the PANSS, which only has a single item for hallucinations across modalities, as opposed to the SAPS, which has auditory-specific items (40).

Interestingly, we observed strong correlation between impaired location MMN and Negative symptoms in CHR, including deterioration in role function, social isolation and decreased experience of emotion, along with dysphoric mood. A similar, but somewhat weaker pattern was observed in schizophrenia, where significant correlations were observed with somatic concern, and passive/apathetic social withdrawal. As compared to other types of stimulus change, changes in stimulus location are processed more rapidly and are more salient than other types of stimulus change (35, 46), suggesting that they play an important role in automatic allocation of attention to salient features of the environment.

### rsFC

In fMRI studies, activation deficits during MMN paradigms are observed in both cortical (10, 11) and subcortical (12) regions, as are deficits in MMN-related visual suppression (11). In addition, deficits are associated with impaired functional connectivity involving auditory, dorsal attention, visual attention, and salience networks (11, 14). To our knowledge, this is the first study to evaluate the interrelationship between MMN generation and internetwork rsFC in CHR subjects as well as the first to evaluate rsFC patterns associated with location MMN.

For location deviants, consistent with a presumed role of MSO in ITD processing, we observed significant correlations involving brainstem regions with larger MMN correlating to increased rsFC between Brainstem and the Dorsal Attention system, but decreased rsFC to Visual and Ventral Attention systems. A limitation of our analyses is that the brainstem ROI was designed for brain parcellation. Our brainstem ROI is not optimized for midbrain nuclei such as superior olive. These correlations nevertheless encourage further investigation of subcortical contributions to impaired location MMN generation across CHR and Sz subjects.

For non-location deviants, increased MMN was associated with increased rsFC between auditory and motor (mouth) networks, consistent with our prior finding (14), as well as between Medial-Parietal and Default networks. By contrast, rsFC involving Salience-Visual networks correlated with lower MMN, consistent with the reciprocal interactions between visual and auditory regions during sensory processing.

### Etiological Implications

At present, our results can be interpreted at either a brainstem or cortical level. At a brainstem level, location MMN based on ITD as in the present study differs from other MMN types based on its dependence on ITD computation within the MSO. The ITD detection circuit is known to be highly dependent upon microglial pruning of glycinergic synapses onto dendrites of MSO neurons (59). To the extent that generalized excessive pruning is involved in the pathogenesis of early schizophrenia, loss in the ability to use interaural timing inputs may index a more general excessive pruning process. Other critical circuit elements for ITD computation within the MSO include a cyclic nucleotide-gated (HCN) channel which generates a cationic inward current, and (2) a dendrotoxin (DTX)-sensitive voltage-gated potassium (Kv1) channel.

Alternatively, to the extent that spatial localization deficits in Sz are observed even to ILD cues, it would argue for a cortical-level impairment particularly in right auditory function that may index analogous deficits in the left hemisphere. Correlations between impaired localization ability and thought disorder would be consistent with models in which impaired hemispheric specialization mediates breakdown in phonological components of language [e.g., (64)]. Overall, further studies comparing location processing to synthesized vs. free-field stimuli are required to better understand the locus of impaired spatial processing in Sz and its functional correlates.

## Limitations

The study is limited by the relatively small sample size, and thus requires replication in larger samples. In addition, the small number of subjects who transitioned to Sz within the follow-up interval prevented assessment of the ability of MMN to differentiate “true” prodromes from phenocopies. The lack of significant pitch and duration MMN deficits in our CHR cohort is consistent with a growing literature suggesting that such MMN types are deficient only within CHR who ultimately transition to Sz. Finally, our location deviant was based only on an ITD cue and did not involve behavioral assessment of localization ability. Thus, processing of other types of location cues and their relationship to behavioral impairments in processes such as auditory object formation needs to be addressed in future studies. fMRI data were collected only for CHR subjects, limiting our ability to compare findings across patient groups. In addition, because a stepwise correlation procedure was used, findings should be considered exploratory and need to be replicated going forward in an independent sample. The subcortical ROIs were also not developed to support rsFC-type studies and may have significantly lower signal-to-noise than cortex. Thus, they need to be refined to better isolate regions potentially involved with subcortical sound localization processes.

## Conclusions

Overall, the present study supports prior demonstrations of impaired MMN in Sz and extends these to include location MMN within an optimized multi-modal paradigm, and its correlation with cognitive symptoms. In addition, it extends MMN findings in the CHR population to include location MMN as well and indicates strong links between impaired location MMN and role function even in CHR individuals who do not ultimately convert to Sz. Impaired localization ability may directly impair role function by interfering with the ability to create auditory “objects” or may index processes such as pruning that may themselves be drivers of impairment.

Because the sample sizes are small, the findings need to be replicated within larger Sz and CHR samples. Nevertheless, the present study argues for investigation of a broad range of MMN types, which probe the auditory system function at varying levels from brainstem through secondary auditory regions and may provide complementary insights regarding neurophysiological abnormalities contributing to impaired function across the Sz and CHR states.

## Data Availability Statement

The raw data supporting the conclusions of this article will be made available by the authors, without undue reservation.

## Ethics Statement

The studies involving human participants were reviewed and approved by the IRBs of New York State Psychiatric Institute/Columbia University and the Nathan Kline Institute for Psychiatric Research IRBs. Written informed consent to participate in this study was provided by the participants' legal guardian/next of kin.

## Author Contributions

PS and DJ participated in study design and implementation. MA performed fMRI analyses. HD collected and analyzed data. CC, RG, and GB contributed to CHR recruitment and assessment. JL-C performed Matlab programming for data analysis. JK and GS contributed to recruitment of Sz subjects. ED and AM participated in ERP data analysis. All authors contributed to the article and approved the submitted version.

## Conflict of Interest

DJ reports Intellectual property for NMDAR agonists in schizophrenia, NMDAR antagonist in depression, fMRI for prediction of ECT response, and ERP biomarkers for diagnosis of mental disorders. Equity in Glytech, AASI, and NeuroRx. Scientific advisory board NeuroRx, Promentis, Consultant payments Autifony, SK Life Sciences, Biogen, Cadence, and Pfizer. RG has consulted for Noble Insights and receives royalties for books from Wipf and Stock and Routledge/Taylor and Francis. GP receives income and equity from Pfizer Inc. through family. AM reports intellectual property for ERP biomarkers for diagnosis of mental disorders. JK reports having received consulting payments within the last 24 months from Alphasights, Charles River Associates, Putnam Associates, Third Bridge, Piper Jaffray, MEDACorp, Parexel, GroupH, Simon Kucher, LifeSci Capital, ECRI Institute, ExpertConnect, Parexel, Schlesinger Group, CelloHealth, Acsel Health, Strafluence, Guidepoint, L.E.K. and System Analytic. He has serves on the MedinCell Psychiatry Advisory Board. He has conducted clinical research supported by the NIMH, Sunovion, Roche, Alkermes, the Stanley Foundation, Cerevance, Takeda, Taisho, Lundbeck, Boehringer Ingelheim, NeuroRX, Teva and Lilly within the last 24 months. JK was a co-investigator on a study that receives lumeteperone and reimbursement for safety testing for an investigator-initiated research from Intra-Cellular Therapies Inc. He owns a small number of shares of common stock from GSK. RG has consulted for Noble Insights and receives royalties for books from Wipf and Stock and Routledge/Taylor and Francis. GP receives income and equity from Pfizer Inc. through family. AM reports intellectual property for ERP biomarkers for diagnosis of mental disorders. The remaining authors declare that the research was conducted in the absence of any commercial or financial relationships that could be construed as a potential conflict of interest.
